# Hypoxia-Inducible Factor–Prolyl Hydroxylase Inhibitor Improves Leukocyte Energy Metabolism in Hereditary Hemorrhagic Telangiectasia

**DOI:** 10.3390/life13081708

**Published:** 2023-08-09

**Authors:** Yves Schild, Jonah Bosserhoff, Freya Droege, Elisabeth Littwitz-Salomon, Joachim Fandrey, Anna Wrobeln

**Affiliations:** 1 Institute of Physiology, University Hospital Essen, University of Duisburg-Essen, 45147 Essen, Germany; yvesleon.schild@uk-essen.de (Y.S.); jonah.bosserhoff@web.de (J.B.); joachim.fandrey@uk-essen.de (J.F.); 2 Department of Otorhinolaryngology, Head and Neck Surgery, University Hospital Essen, University of Duisburg-Essen, 45147 Essen, Germany; freya.droege@uk-essen.de; 3 Institute for Virology, University Hospital Essen, University of Duisburg-Essen, 45147 Essen, Germany; elisabeth.littwitz-salomon@uk-essen.de; 4 Institute for Translational HIV Research, University Hospital Essen, University of Duisburg-Essen, 45147 Essen, Germany

**Keywords:** hypoxia-inducible factor, HIF, leukocytes, PBMC, hereditary hemorrhagic telangiectasia, HHT, hypoxia, Roxadustat, HIF–prolyl hydroxylase inhibitor

## Abstract

The interplay between hypoxia-inducible factors (HIFs) and transforming growth factor beta (TGF-β) is critical for both inflammation and angiogenesis. In hereditary hemorrhagic telangiectasia (HHT), we have previously observed that impairment of the TGF-β pathway is associated with downregulation of HIF-1α. HIF-1α accumulation is mandatory in situations of altered energy demand, such as during infection or hypoxia, by adjusting cell metabolism. Leukocytes undergo a HIF-1α-dependent switch from aerobic mitochondrial respiration to anaerobic glycolysis (glycolytic switch) after stimulation and during differentiation. We postulate that the decreased HIF-1α accumulation in HHT leads to a clinically observed immunodeficiency in these patients. Examination of HIF-1α and its target genes in freshly isolated peripheral blood mononuclear cells (PBMCs) from HHT patients revealed decreased gene expression and protein levels of HIF-1α and HIF-1α-regulated glycolytic enzymes. Treatment of these cells with the HIF–prolyl hydroxylase inhibitor, Roxadustat, rescued their ability to accumulate HIF-1α protein. Functional analysis of metabolic flux using a Seahorse FX extracellular flux analyzer showed that the extracellular acidification rate (indicator of glycolytic turnover) after Roxadustat treatment was comparable to non-HHT controls, while oxygen consumption (indicator of mitochondrial respiration) was slightly reduced. HIF stabilization may be a potential therapeutic target in HHT patients suffering from infections.

## 1. Introduction

Of all rare diseases hereditary hemorrhagic telangiectasia (HHT), also known as Rendu–Osler–Weber syndrome, is quite common, with a prevalence of around 1:5000, i.e., HHT affects worldwide ~1.6 million people and ~85,000 people in Europe alone [1]. HHT patients suffer from dilated abnormal vascular structures, often leading to recurrent hemorrhages. One of the clinical manifestations of this rare inherited disease is the occurrence of multisystemic arteriovenous malformations, particularly in the liver, lungs, intestine, and brain [2]. The mutations affect genes in the transforming growth factor beta (TGF-β)/bone morphogenetic protein 9 (BMP9) pathway, resulting in impaired signal transduction [3]. As a regulatory cytokine, TGF-β is of major function in several pivotal cellular processes, such as cell growth, apoptosis, smooth muscle cell differentiation, vascular remodeling and the immune response [4]. In addition to the commonly observed vascular-related symptoms, lately, physicians observed that HHT patients suffer from a compromised immune system, resulting in increased rates of sinusitis, pneumonia, and urinary tract infections. Statistics reveals that sepsis is a leading cause of death in HHT patients [5,6,7,8,9,10]. The impaired immune response was demonstrated in both human and mouse studies [6,11,12,13]. In addition to TGF-β, the transcription factor hypoxia-inducible factor (HIF) performs similar functions in cells, and an interplay between the two factors was previously demonstrated [14,15,16,17]. HIFs are oxygen sensitive transcription factors consisting of an alpha subunit and a nuclear beta subunit. Cells express both subunits constitutively. However, under normoxic conditions, the prolyl hydroxylases use elemental oxygen to attach a hydroxyl group to HIF-α [18]. This modification leads to ubiquitination of the HIF-α subunit and, subsequently, to the degradation in the proteasome. Hypoxia inhibits this process due to the reduced availability of elemental oxygen in the cytosol [19]. The main task of HIF is cellular oxygen sensing, followed by the subsequent adaptation to hypoxic conditions via induction of specific targeted genes involved in vascularization, angiogenesis, metabolism and cell survival [20]. In addition to accumulation under hypoxic conditions, HIF accumulates in situations of increased energy demand, e.g., during an infection, and ensures the proper functioning of leukocytes by adjusting cell-metabolism [21]. Upon stimulation, several leukocytes undergo a HIF-1α-dependent switch on the energy metabolism from the more efficient but slower aerobic mitochondrial respiration that is fed by fatty acids, amino acids and ketone bodies, to a faster but strictly glucose-dependent anaerobic glycolysis (glycolytic switch) [22,23]. Several studies have shown immune cell dysfunction in the absence of HIFs [24,25]. Recently, our group quantified, for the first time ever, the expression of HIF-1α and HIF target genes in whole blood samples from HHT patients [26]. We showed a significant reduction in the expression of *HIF1A* gene, HIF-1α protein and HIF-1α target genes in whole blood of HHT patients. This led us to hypothesize that the observed dysregulation of HIF may be the reason for the clinically observed immune dysfunction in HHT patients.

In this study, for the first time, we isolated leukocytes from HHT patients to analyze their HIF expression pattern. Based on these findings, we intervened by treating the isolated cells with the pharmaceutical prolyl hydroxylase inhibitor, Roxadustat, to study the cell response to HIF accumulation. We analyzed the extracellular acidification rate as an indicator of glycolytic function and the oxygen consumption rate as an indicator of mitochondrial respiration of HHT leukocytes simultaneously in the presence and absence of Roxadustat (pharmacological PHD inhibition/HIF-1a stabilization) using a Seahorse extracellular flux analyzer.

## 2. Methods

### 2.1. Selection of Participants

Adult HHT patients who fulfilled at least three out of the four Curaçao criteria and/or had a positive genetic test were included in this prospective study [2,27]. In all cases, blood samples were collected from patients’ peripheral arm veins. All experiments were performed with blood from a non-HHT control group. The study was approved by the Ethics Committee of the University Duisburg-Essen (20-9162-BO). Participants were informed and provided written consent in accordance with the Declaration of Helsinki. The study is registered at clinicaltrials.gov (ID NCT04469517).

### 2.2. RNA Isolation from Whole Blood

Whole blood RNA was isolated using the PAXgene^®^ blood RNA collection system (Qiagen, Mississauga, ON, Canada), according to the manufacturer’s protocol. A total of 2.5 mL of whole blood was collected from HHT patients or non-HHT controls into PAXgene^®^ blood RNA tubes (Qiagen, Mississauga, ON, Canada) and stored at −20 °C.

### 2.3. PBMC Isolation and Cultivation

PBMCs were isolated using SepMate^TM^ tubes (STEMMCELL Technologies Inc., Vancouver, BC, Canada), according to the manufacturer’s instructions. Lymphoprep^TM^ density gradient medium (STEMMCELL Technologies Inc., Vancouver, BC, Canada) was used for cell isolation.

Isolated PBMCs were suspended in RPMI 1640 medium (Gibco, BioWhittaker, Verviers, Belgium) supplemented with 10% (*v*/*v*) fetal calf serum (Sigma, St. Louis, MO, USA), penicillin (100 U/mL), and streptomycin (100 μg/mL) and transferred to a 6-well plate at a concentration of 4 million cells/well and allowed to rest overnight at 37 °C in a humidified atmosphere (5% CO_2_). On the day of the experiment, PBMCs were incubated under humidified conditions with 21% O_2_ and 5% CO_2_.

### 2.4. Pharmacological Stabilization of HIF-1α

Roxadustat (Cayman Chemical, Hamburg, Germany) (30 µM in DMSO) was administered for 24 h at 37 °C, 21% O_2_, 5% CO_2_. The vehicle control consisted of equivalent concentrations of DMSO.

### 2.5. RNA Isolation and Gene Expression Analysis by Real-Time PCR

Total RNA was isolated from PBMCs using the NucleoSpin RNA Kit (Macherey–Nagel, Dueren, Germany), according to the manufacturer’s protocol. Gene quantification was performed by real-time PCR (RT-PCR) using SYBR green fluorescent dye (Eurogentec, Verviers, Belgium) and the CFX96TM Real-Time System (Bio-Rad Laboratories GmbH, Munich, Germany). We reverse transcribed 200 ng of total RNA into cDNA, which was amplified with gene-specific primers (Table 1) and normalized to ACTB (actin). Primer specificity was checked by Primer-BLAST and confirmed by size analysis of the PCR amplicons. Expression was calculated using the 2^−ΔΔCT^ method for statistical analysis and set as an induction relative to the respective non-HHT controls.

### 2.6. Protein Isolation and Western Blot

PBMCs were lysed with a lysis buffer (150 mM NaCl, 20 mM Tris (pH 7.5), 5 mM EDTA and 1% NP-40) containing 10% freshly added proteinase inhibitor (Roche Diagnostics, Basel, Switzerland) for 30 min on wet ice. After centrifugation at 10,000× *g* for 5 min at 4 °C, the PBMC supernatant was collected and stored at −80 °C until further use. Bio-Rad protein assay reagent (Bio-Rad, Munich, Germany) was used to determine protein concentration. In total, 60 µg of each cell lysate was boiled in sodium dodecyl sulfate (SDS) buffer and subjected to SDS-polyacrylamide gel electrophoresis (120 min, 80 V). The samples were then transferred to a polyvinylidene difluoride membrane (peqlab-VWR, Darmstadt, Germany) using the Trans-Blot^®^ Turbo™ Transfer System (Bio-Rad, Munich, Germany). Membranes were blocked in 5% of milk in Tris-buffered saline containing 0.05% Tween. Primary antibodies against β-actin (loading control: 1:1000; Sigma Aldrich, St. Louis, MO, USA) and HIF-1α (1:500; BD Transduction Laboratories™, Franklin Lakes, NJ, USA) were used in 5% of milk in Tris-buffered saline, followed by incubation with a horseradish peroxidase-conjugated anti-rabbit or anti-mouse secondary antibody. Chemiluminescence was detected using SuperSignal™ West Femto Maximum Sensitivity Substrate (Thermo Scientific, Waltham, MA, USA) and visualized in Fusion FX (Vilber Smart Signaling, Marne-la-Vallée Cedex 3, France). ImageJ software was used for semi-quantification.

### 2.7. Metabolic Flux Analysis

Oxygen consumption rate (OCR, pmoles/min) and extracellular acidification rate (ECAR, mpH/min) were determined using a Seahorse XFe Extracellular Flux Analyzer (Agilent Technologies, Santa Clara, CA, USA), according to the manufacturer’s protocol. The wells of an 8-well polystyrene Seahorse plate were treated with Cell-Tak (Corning, NY, USA) to ensure adherence of PBMCs to the plate. PBMCs treated with either Roxadustat (30 µM, 24 h) or DMSO (vehicle control, equivalent volume to Roxadustat, 24 h) were plated at a density of 400,000 cells per well. Measurements of OCR and ECAR after addition of the inhibitors 15 µM Oligomycin (Santa Cruz, Dallas, TX, USA), 12.5 µM FCCP (Santa Cruz, Dallas, TX, USA), 10 µM Rotenone/Antimycin A (Sigma, Darmstadt, Germany), and 50 mM 2-Deoxyglucose (2DG, Fisher Scientific, Waltham, MA, USA), allowed the calculation of adenosine triphosphate (ATP)-production, respiratory capacity, max respiration, basal glycolysis and glycolytic capacity.

Normalization was performed using the combined bright field imaging and image analysis procedure PIXI. PIXI is an R-integrated pixel intensity analysis tool developed and validated for the use on PBMCs by Janssen et al. The tool was assembled from EBImage packages by Pau et al. 2010 from the Bioconductor program library [28,29].

After the flux assay, bright field images of each well were captured using a Zeiss Axiovert with a 4× objective. Subsequent image analysis was performed in R using the PIXI-tool with the following steps: A Gaussian Blur Low Pass Filter was used to create a background image, which was subtracted from the original image, inverted and cropped to an appropriate size. For the processed images, the total pixel intensity was determined, from which the normalization factor for each individual well was derived.

### 2.8. Statistics

GraphPad Prism 8.4.3. (GraphPad Software Inc., La Jolla, CA, USA) was used for statistical analysis. After elimination of outliers (ROUT-test), the groups were analyzed for Gaussian distribution using the D’Agostino-Pearson test. Unpaired *t*-test was used for Gaussian distributed data and Mann–Whitney U test for non-Gaussian distributed data. A two-way ANOVA was used for metabolic flux analysis. Statistical significance is shown as ns (not significant), * (*p* < 0.05), ** (*p* < 0.01), *** (*p* < 0.001) or **** (*p* < 0.0001).

## 3. Results

### 3.1. HIF-1 Target Genes Related to Metabolism Are Downregulated in HHT Whole Blood

**Figure 1:**
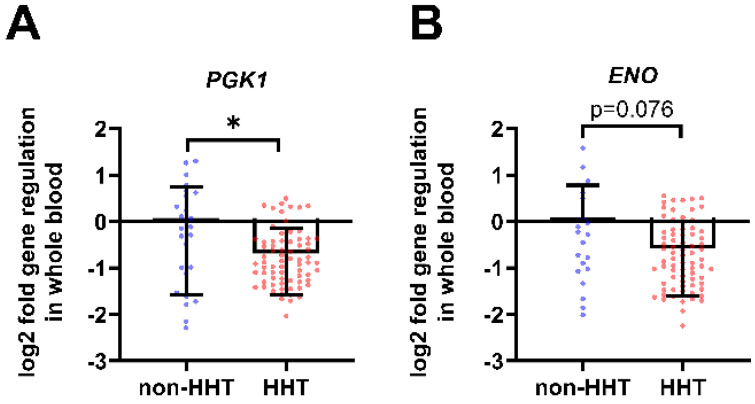
In whole blood from HHT patients, HIF-1 metabolic target genes are downregulated. Whole blood gene expression of glycolysis related HIF-1 target genes phosphoglycerate kinase 1 (*PGK1*) shown in (**A**) and enolase (*ENO*) shown in (**B**) were downregulated in HHT patients (*n* = 73) compared to the non-HHT controls (*n* = 25). Data evaluation: Mann–Whitney U test, * (*p* < 0.05); mean ± SD.

Whole blood mRNA analysis of HHT patients and non-HHT controls showed a decrease in HIF-1 target genes related to glycolysis. Phosphoglycerate kinase 1 (*PGK1*) gene expression was significantly reduced in HHT whole blood compared to non-HHT controls (Figure 1A). The Enolase (*ENO*) gene expression was decreased in HHT whole blood compared to non-HHT controls (Figure 1B).

### 3.2. HIF1A and Metabolic Target Gene Expression Is Decreased in HHT PBMCs

**Figure 2:**
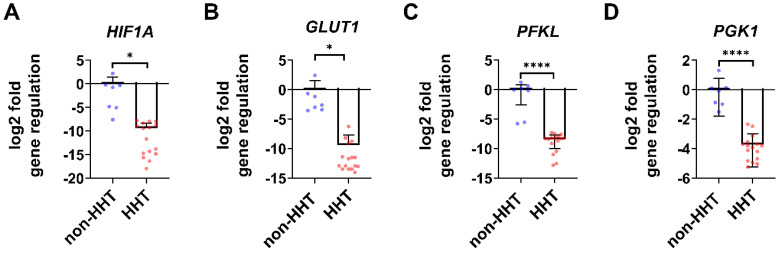
In isolated HHT PBMCs *HIF1A* gene expression as well as metabolic target gene expression are downregulated. Isolated PBMCs showed a significant reduction of *HIF1A* gene expression (**A**) in HHT patients. HIF-1 target genes related to metabolism were downregulated in HHT patients: glucose transporter 1 (*GLUT1*) (**B**), phosphofructokinase (*PFKL*) (**C**) and phosphoglycerate kinase 1 (*PGK1*) (**D**). Data evaluation: unpaired *t*-test. * (*p* < 0.05), **** (*p* < 0.0001); mean ± SD (HHT: *n* = 16, non-HHT: *n* = 7).

*HIF1A* gene expression is significantly decreased in isolated HHT PBMCs compared to non-HHT PBMCs (Figure 2A). In addition, the analyzed glycolytic HIF-1α target genes glucose transporter 1 (*GLUT1*; Figure 2B), phosphofructokinase (*PFKL*; Figure 2C) and phosphoglycerate kinase 1 (*PGK1*; Figure 2D) were significantly reduced in HHT PBMCs compared to non-HHT controls.

### 3.3. HIF-1α Protein Is Reduced in HHT PBMCs but Can Be Pharmacologically Normalized

**Figure 3:**
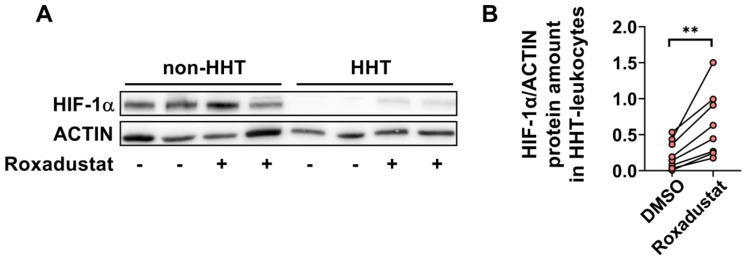
HIF-1α protein expression is downregulated in HHT PBMCs but stabilized through pharmacological treatment with Roxadustat. (**A**) Representative Western blot of HIF-1α protein (120 kDa) and ACTIN protein (42 kDa, as loading control) in HHT and non-HHT PBMCs. (**B**) Paired analysis of the quantification of HIF-1α protein in HHT PBMCs before and after Roxadustat treatment. Data evaluation: paired *t*-test, ** (*p* < 0.005).

We detected a reduced amount of HIF-1α protein in HHT PBMCs compared to non-HHT controls. The accumulation of HIF-1α protein in HHT and non-HHT PBMCs increased after treatment with 30 µM Roxadustat for 24 h (Figure 3A). Quantification of the HIF-1α protein levels showed a significant increase after Roxadustat treatment in HHT PBMCs (Figure 3B).

### 3.4. Metabolic Capacity Is Reduced in HHT PBMCs

#### 3.4.1. HHT PBMCs Have Reduced Mitochondrial Metabolic Activity, Which Can Be Recovered by HIF Stabilization

Comparison of the mitochondrial metabolic activity of non-HHT and HHT PBMCs by Seahorse flux analysis in combination with respiratory chain complex inhibition revealed a slightly lower basal oxygen consumption rate (OCR) in native HHT PBMCs, compared to native non-HHT PBMCs. Inhibition of the ATP-synthase reduced the OCR in both groups. Uncoupling of oxidative phosphorylation revealed a lower respiratory capacity in HHT PBMCs compared to non-HHT PBMCs. Inhibition of the electron transfer to oxygen resulted in decreased maximal respiration in HHT PBMCs compared to non-HHT controls.

**Figure 4:**
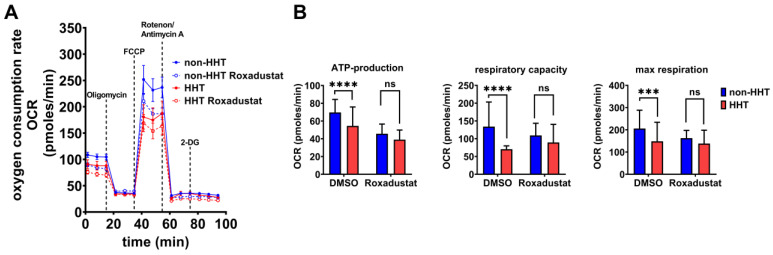
Mitochondrial respiration capacity is impaired in HHT PBMCs but recoverable through pharmacological stabilization of HIF-1α protein. (**A**) The oxygen consumption rate (OCR) of HHT PBMCs (red, dashed = Roxadustat treatment) and non-HHT PBMCs (blue, dashed = Roxadustat treatment). (**B**) Calculated ATP-production, respiratory capacity and maximal respiration of HHT- and non HHT-PBMCs with and without Roxadustat treatment. Data evaluation: *n* = 6; 2 way ANOVA; ns (not significant), *** (*p* < 0.001), **** (*p* < 0.0001); mean ± SD.

Roxadustat treatment reduced basal OCR in both non-HHT and HHT PBMCs, with a stronger effect in HHT PBMCs. Uncoupling of oxidative phosphorylation by FCCP under Roxadustat treatment decreased OCR in both non-HHT and HHT PBMCs, again with a stronger effect in HHT PBMCs (Figure 4A).

Significant reductions in calculated ATP production, respiratory capacity and maximal respiration were observed in HHT PBMCs without Roxadustat treatment. After the administration of Roxadustat, ATP production decreased in both groups with a smaller effect in HHT cells, and the significant difference between the groups was no longer observed. Respiratory capacity showed a slight increase in HHT PBMCs after Roxadustat treatment, whereas non-HHT PBMCs showed a slight decrease, so that there was no significant difference between the groups. Maximal respiration was barely affected in HHT PBMCs after Roxadustat treatment but decreased in non-HHT PBMCs; the difference between non-HHT and HHT was no longer significant (Figure 4B).

#### 3.4.2. Glycolytic Metabolism Is Reduced in HHT PBMCs but Recoverable through Pharmacological Stabilization of the HIF-1α Protein

**Figure 5:**
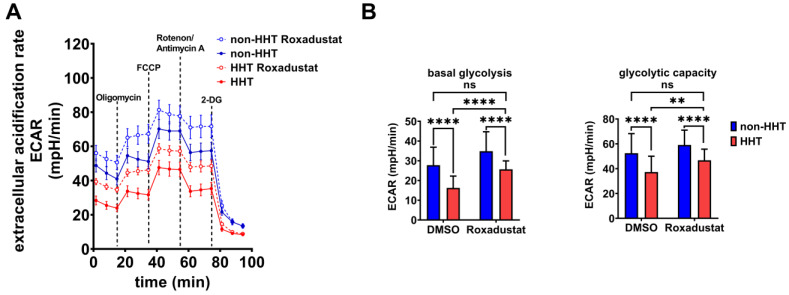
Glycolytic metabolism is reduced in HHT PBMCs but is recovered through pharmacological stabilization of HIF-1α protein. (**A**) Extracellular acidification rate (ECAR) of HHT PBMCs (red, dashed = Roxadustat treatment) compared to non-HHT PBMCs (blue, dashed = Roxadustat treatment). (**B**) Calculated basal glycolysis and glycolytic capacity in HHT- and non-HHT PBMCs before and after Roxadustat treatment. Data evaluation: *n* = 6; 2 way ANOVA; ns (not significant), ** (*p* < 0.01), **** (*p* < 0.0001); mean ± SD.

The extracellular acidification rate (ECAR) is a parameter to determine glycolytic lactate production in cells. The basal ECAR indicates how efficiently cells can feed pyruvate into the citric acid cycle. Blocking the respiratory chain with oligomycin, FCCP, rotenone and antimycin allows for the determination of the maximal glycolytic capacity. HHT PBMCs showed reduced basal glycolysis and glycolytic capacity that could be rescued via Roxadustat administration (Figure 5A). Although Roxadustat treatment did not reach glycolytic activity of non-HHT PBMCs, HHT PBMCs reached the level of untreated PBMCs and thus the healthy state (Figure 5B). Therefore, Roxadustat treatment fully compensated for the reduction of glycolysis caused by HHT. The administration of 2-DG determines the background acidification resulting from all cellular processes except glycolysis.

## 4. Discussion

We have identified (I) reduced HIF-1α gene expression and protein levels in isolated PBMCs from HHT patients, (II) reduced HIF-1α target gene expression of genes related to metabolism, (III) impaired metabolic capacity of HHT PBMCs, and (IV) the possibility of enhancing metabolism by pharmacological HIF-1α stabilization. Publications indicate that HHT patients suffer from an impaired immune response with an altered immune cell composition, impaired ability of immune cells to migrate into tissues, and impaired physiological function [6,9,13,30].

The reduced expression of HIF-1α in leukocytes of HHT patients may explain some observations of immune defects in HHT patients [26]. Phosphoglycerate kinase 1 (PGK1) catalyzes the removal of phosphate from 1, 3-bisphosphoglycerate to ADP to form 3-phosphoglycerate and ATP as a sub-step of glycolysis. By controlling ATP and 3-PG levels, PGK1 plays an important role in coordinating energy production with biosynthesis and redox balance [31]. Since *PGK1* expression reflects the cellular glycolytic activity to some extent, as demonstrated by Li et al., we hypothesize that energy production via glycolysis may be impaired in HHT blood cells [32]. The reduction of *PGK1* in leukocytes observed in our study may lead to reduced ATP supply during inflammation (Figure 1). Furthermore, since PGK1 is a stimulatory factor for neutrophils, we assume that PGK1 reduction may—at least in part–explain the impaired physiological function of neutrophils in HHT patients, as shown by Droege et al. [13,33]. The enzyme enolase (ENO) catalyzes the dehydration of 2-phospho-D-glycerate to phosphoenolpyruvate in the forward or catabolic direction in the second half of the glycolytic pathway. Reduced *ENO* expression could likewise not only contribute to an impaired energy supply, but also explain the documented increased risk of thrombosis in HHT patients, although HHT is not a coagulation disorder [34]. Leukocytes such as neutrophils, B cells, T cells, and monocytes express ENO as a plasminogen receptor on their surface, and the binding of ENO might be required for plasminogen activation and plasmin generation [35,36,37,38,39]. Additionally, ENO could play a role in monocyte recruitment in inflammatory lung disease and thus contribute to the impaired immune cell function in HHT patients [40]. Glucose transporter 1 (GLUT1) inhibition reduced CD4+ T cell proliferation and reduced IFN-γ secretion by 20% in a mouse model study by Chen et al. In addition, TNF secretion from macrophages was reduced by 27% [41]. Previously, GLUT1 was shown to be selectively essential for CD4+ T cell activation and effector function [42]. The reduction in *GLUT1* expression we observed may be related to the impaired physiological function of leukocytes in HHT patients. The glycolytic rate limiting phosphofructokinase (*PFKL*) is essential for ATP generation, autophagy and redox balance in rheumatoid arthritis T cells [43].

Since most of the glycolysis-related genes we examined were reduced, we focused on the metabolism of the PBMCs in the following. Like HHT whole blood, isolated HHT PBMCs showed reduced HIF-1α gene expression and protein levels (Figure 2 and Figure 3). PBMCs typically consist of 70–85% T lymphocytes, 5–10% B lymphocytes, 5–20% natural killer cells, 10–20% monocytes, and 1–2% dendritic cells [44,45]. Chimeric mice with HIF-1α-deficient B and T cells showed increased tissue damage and autoimmunity, and mice with conditional knockout of HIF-1α in granulocytes, monocytes and macrophages showed impaired myeloid cell infiltration and activation in vivo, highlighting the important role of HIF-1α in immune responses [25,46]. The reduced expression of HIF-1α can explain the observed reduction of key metabolic genes in HHT PBMCs (Figure 2). Cramer et al. earlier postulated that HIF-1α is essential for the regulation of glycolytic capacity in myeloid cells and that in the absence of HIF-1α, the cellular ATP pool is drastically reduced [28]. In their work, the metabolic defect due to HIF-1α deletion in myeloid cells resulted in profound impairment of myeloid cell aggregation, motility, invasiveness, and bacterial killing [46].

We observed reduced glycolytic activity and mitochondrial respiration in HHT PBMCs (Figure 4 and Figure 5). If HIF-1α is the cause of impaired glycolytic activity and therefore immune deficiency in HHT patients, then enhancing HIF will lead to improved energy metabolism and potentially an improved immune response in HHT patients. We found that Roxadustat, which inhibits HIF hydroxylation through prolyl hydroxylases and degradation through the proteasome and thus stabilizes HIF-1α protein, significantly increased HIF-1α protein in PBMCs from HHT patients (Figure 3). Although stabilization of HIF-1α did not result in further reduction of oxidative phosphorylation, we observed a significant increase in glycolysis in comparison to untreated PBMCs unaffected by HHT. Roxadustat was not as effective in HHT PBMCs as in the control PBMCs but compensated for the reduction caused by HHT. Obviously, the underlying mechanism causing reduced HIF-1α expression in HHT is more complex and is under further investigation.

At this stage, we can conclude that the reduced HIF-1α level in HHT has a significant impact on the metabolism of leukocytes, but is most likely not the sole reason for the impaired function of immune cells in HHT. However, Roxadustat is officially approved for clinical use in both China and Europe for the treatment of patients with renal anemia to increase expression of the HIF target gene erythropoietin [47]. Thus, one may envision the off-label use of Roxadustat to benefit HHT patients in two ways: first, by enhancing the immune response, and second, by reducing hemorrhagic-related anemia, as HIF-prolyl hydroxylase inhibition actually increases iron availability by increasing both iron metabolism and iron uptake [48,49,50]. The clinical use of HIF-prolyl hydroxylase inhibitors, such as Roxadustat, may be a promising approach to address several defects related to HHT.

## Figures and Tables

**Table 1:** Human primer sequences.

Gene	5′ Primer	3′ Primer
*ACTB*	TCACCCACACTGTGCCCATCTACGA	CAGCGGAACCGCTCATTGCCAATGG
*PGK1*	TGGACGTTAAAGGGAAGCGG	GCTCATAAGGACTACCGACTTGG
*ENO*	GCCGTGAACGAGAAGTCCTG	ACGCCTGAAGAGACTCGGT
*HIF1A*	TCACTGGGACTATTAGGCTCAGGT	CTCCATTACCCACCGCTGAA
*GLUT1*	TCTGGCATCAACGCTGTCTT	CTAGCGCGATGGTCATGAGT
*PFKL*	GCTGGGCGGCACTATCATT	TCAGGTGCGAGTAGGTCCG

Primer sequences of specific PCR products used for RNA quantifications of PBMCs via RT-PCR.

## Data Availability

Requests for data, methods or materials should be addressed to A.W.
